# Chicago Society of Physicians and Surgeons

**Published:** 1875-07-01

**Authors:** E. Warren Sawyer


### CHICAGO SOCIETY OF PHYSICIANS AND SURGEONS.

### REGULAR MEETING, JUNE 14TH, 1875.

*Reported by E. Warren Sawyer, M.D.*

VICE-PRESIDENT Dr. Hamill in the chair.

Dr. Warn read an abstract of Dr. Byford's paper on the treatment of uterine fibroid tumors by the use of ergot. The paper contained an interesting consideration of the kind of tumor which was most amenable to treatment, the form of ergot most eligible, and the best manner of administering the drug.

Those tumors situated most nearly to the mucous surface are the most favorable for this treatment, though in one instance which was frequently examined by Dr. Byford, a polypus, with a long pedicle attached to the upper part of the cervix, but projecting into the vagina, was seen to decrease rapidly in size under the influence of ergot.

Squibb's fld. extract of ergot is the most reliable for internal use, and the solution of the watery extract of ergot in glycerine, as suggested by Dr. Way, is the most eligible mixture we now possess for hypodermic injections.

Dr. Byford selects the internal administration of the drug; giving a fld. drachm, three times daily, the treatment to be kept up for months, according to circumstances. In some cases, the combining of the drug with belladonna increases the good effect. The hypodermic use of the drug is not usually painful, nor do abscesses commonly follow; thus Hildebrandt says that in one thousand subcutaneous injections not one abscess resulted.

The paper contained an account of many cases of this use of ergot, some under Dr. Byford's observation and others under the care of various observers throughout the country, who had communicated the results to him. In all, 103 cases had been reported: in twenty-three instances the tumors disappeared entirely; in thirty-eight it was diminished in size; in nineteen the hæmorrhage was arrested and the patient relieved, while only twenty-one resisted entirely the effects of the treatment.

Dr. H. A. Johnson reported a case of intestinal obstruction, in which after death a gall stone was found blocking up the small intestine.

Dr. A. Reeves Jackson exhibited to the society two sizes of a new vaginal speculum, and made some remarks upon the same. The instrument is a single-bladed Sim's speculum, the blade being flattened somewhat and shorter. It is designed to be introduced into the vagina over the posterior commissure, and with it the perineum dragged backwards; in this way the vagina becomes inflated and, because the patient is upon her back, the os and cervix are low down and very near the vulva. Over Sim's instrument it has the advantage that the patient is upon her back (in the lithotomy position), which is much more favorable for anæsthesia. Sargent, of this city, has made the instrument of a new alloy, which has the appearance of gold, admits of a fine polish, and which is almost unaffected by acids.

